# A subclinical high tricuspid regurgitation pressure gradient independent of the mean pulmonary artery pressure is a risk factor for the survival after living donor liver transplantation

**DOI:** 10.1186/s12876-018-0793-z

**Published:** 2018-05-15

**Authors:** Yosuke Saragai, Akinobu Takaki, Yuzo Umeda, Takashi Matsusaki, Tetsuya Yasunaka, Atsushi Oyama, Ryuji Kaku, Kazufumi Nakamura, Ryuichi Yoshida, Daisuke Nobuoka, Takashi Kuise, Kosei Takagi, Takuya Adachi, Nozomu Wada, Yasuto Takeuchi, Kazuko Koike, Fusao Ikeda, Hideki Onishi, Hidenori Shiraha, Shinichiro Nakamura, Hiroshi Morimatsu, Hiroshi Ito, Toshiyoshi Fujiwara, Takahito Yagi, Hiroyuki Okada

**Affiliations:** 1 Department of Gastroenterology and Hepatology, Okayama University Graduate School of Medicine, Dentistry and Pharmaceutical Sciences, 2-5-1 Shikata-cho, Kita-ku, Okayama, 700-8558 Japan; 2 Department of Gastroenterological Surgery, Transplant and Surgical Oncology, Okayama University Graduate School of Medicine, Dentistry and Pharmaceutical Sciences, 2-5-1 Shikata-cho, Kita-ku, Okayama, 700-8558 Japan; 3 Department of Anesthesiology and Resuscitology, Okayama University Graduate School of Medicine, Dentistry and Pharmaceutical Sciences, 2-5-1 Shikata-cho, Kita-ku, Okayama, 700-8558 Japan; 4 Department of Cardiovascular Medicine, Okayama University Graduate School of Medicine, Dentistry and Pharmaceutical Sciences, 2-5-1 Shikata-cho, Kita-ku, Okayama, 700-8558 Japan

**Keywords:** Hepatopulmonary syndrome, Living donor related liver transplantation, Portopulmonary hypertension, Tricuspid regurgitation pressure gradient

## Abstract

**Background:**

Portopulmonary hypertension (POPH) is characterized by pulmonary vasoconstriction, while hepatopulmonary syndrome (HPS) is characterized by vasodilation. Definite POPH is a risk factor for the survival after orthotopic liver transplantation (OLT), as the congestive pressure affects the grafted liver, while subclinical pulmonary hypertension (PH) has been acknowledged as a non-risk factor for deceased donor OLT. Given that PH measurement requires cardiac catheterization, the tricuspid regurgitation pressure gradient (TRPG) measured by echocardiography is used to screen for PH and congestive pressure to the liver. We investigated the impact of a subclinical high TRPG on the survival of small grafted living donor liver transplantation (LDLT).

**Methods:**

We retrospectively analyzed 84 LDLT candidates. Patients exhibiting a TRPG ≥25 mmHg on echocardiography were categorized as potentially having liver congestion (subclinical high TRPG; *n* = 34). The mean pulmonary artery pressure (mPAP) measured after general anesthesia with FIO_2_0.6 (mPAP-FIO_2_0.6) was also assessed. Patients exhibiting pO_2_ < 80 mmHg and an alveolar-arterial oxygen gradient (AaDO_2_) ≥ 15 mmHg were categorized as potentially having HPS (subclinical HPS; *n* = 29). The clinical course after LDLT was investigated according to subclinical high TRPG.

**Results:**

A subclinical high TRPG (*p* = 0.012) and older donor age (*p* = 0.008) were correlated with a poor 40-month survival. Although a higher mPAP-FIO_2_0.6 was expected to correlate with a worse survival, a high mPAP-FIO_2_0.6 with a low TRPG was associated with high frequency complicating subclinical HPS and a good survival, suggesting a reduction in the PH pressure via pulmonary shunt.

**Conclusion:**

In cirrhosis patients, mPAP-FIO_2_0.6 may not accurately reflect the congestive pressure to the liver, as the pressure might escape via pulmonary shunt. A subclinical high TRPG is an important marker for predicting a worse survival after LDLT, possibly reflecting congestive pressure to the grafted small liver.

## Background

End-stage liver cirrhosis is often complicated with pulmonary capillary disorders. The most commonly found pulmonary vascular complication is hepatopulmonary syndrome (HPS), in which the pulmonary arteries dilate, resulting in arterial deoxygenation (an increased alveolar-arterial oxygen gradient [AaDO_2_] with or without hypoxemia) [1]. Although not frequent, occasional reports of portopulmonary hypertension (POPH), in which the pulmonary arteries thicken, resulting in pulmonary hypertension (PH), have been described. The criteria for diagnosing POPH are PH (defined by a mean pulmonary artery pressure [mPAP] > 25 mmHg at rest via right heart catheterization) and high pulmonary vascular resistance without complicating left heart failure. These criteria indicate that POPH is a form of pulmonary hypertension not due to left ventricular heart failure in portal hypertensive patients. The prevalence of HPS in patients with cirrhosis ranges from 10 to 30%, while that of POPH ranges from 5 to 10% [2]. Both of these complications significantly worsen the prognosis and quality of life [3].

Given that HPS can be resolved after orthotopic liver transplantation (OLT) even when hypoxemia is severe (PaO_2_ < 50 mmHg), the implications for OLT are understood to apply not only with deceased donor LT (DDLT) but also living donor related LT (LDLT). However, the post-OLT course of POPH-complicated patients is often unsatisfactory, and severe (mPAP > 45 to 50 mmHg) patients are considered absolutely contraindicated for OLT. The International Liver Transplant Society Practice Guidelines indicate that, unlike HPS, there are no data to support the concept that POPH (treated or untreated) should be an indication for OLT [4]. Several clinical studies have suggested that if mPAP is < 35 mmHg, then the perioperative mortality does not increase [5, 6]. Furthermore, the Practice Guidelines recommend that patients with mPAP < 35 mmHg be indicated for OLT, and PA-targeted therapy should be initiated in patients with mPAP ≥35 mmHg [4]. However, almost all of the patients in these articles had received DDLT, and no such analyses have been performed for LDLT patients. Given that left or right lobe LDLT grafts are smaller than DDLT grafts, PH-induced hepatic venous pressure might result in a strong congestive impact on the LDLT grafted liver.

The objective of the present study was to investigate the impact of cirrhosis-related cardio-pulmonary complications on the LDLT survival. Although patients with confirmed POPH and HPS are rare, those with mild pulmonary hypertension and mild HPS are more common. Given that right heart catheterization is not performed for all patients during the pre-operative investigation, the tricuspid regurgitation pressure gradient (TRPG) measured by transthoracic echocardiography (TTE) was adopted to estimate the PH-related congestive pressure in the transplanted liver. The peak systolic pressure gradient between the right ventricle (RV) and the right atrium (RA) can be estimated using the peak systolic TRPG [7]. The TRPG is calculated from the peak velocity of the tricuspid regurgitation (TR) measured by continuous-wave Doppler echocardiography using the modified Bernoulli equation [8].

The risk factors associated with the 3-month, 1-year, and 40-month survival were investigated.

## Methods

### Subjects

The study group consisted of 84 retrospectively analyzed liver cirrhosis patients who received LDLT at our hospital (Table 1). All patients were recruited at the Clinic of Gastroenterology and Hepatology, Okayama University Hospital, from December 2008 to September 2015, indicating that all patients were followed for longer than 1 year. Definitely diagnosed HPS before OLT was noted in only one patient, and POPH before OLT was also noted in only one patient. The definitively diagnosed POPH patients received specific treatment with prostaglandin I2 and showed a normal range of TRPG and mPAP at LDLT.

**Table 1:** Patient characteristics

	All	Subclinical no-HPS vs. Subclinical HPS			Low/normal-TRPG vs. Subclinical high TRPG		
		Subclinical no-HPS	Subclinical HPS	*p*	Low/normal-TRPG	Subclinical high-TRPG	*p*
n	84	55	29		42	34	–
Age (years)	57 (51–61)	58 (52–61)	55 (49–63)	0.716	57 (50–61)	57 (51–61)	0.593
Donor age (years)	39 (32–52)	40 (32–55)	38 (29–46)	0.374	39 (31–52)	40 (31–47)	0.732
Male (%)	45 (53)	31 (56)	14 (48)	0.479	24 (57)	18 (52)	0.714
BMI	23.4 (21.8–26.9)	23.8 (21.7–26.2)	23.3 (22.0–28.3)	0.686	22.6 (21.7–26.1)	23.9 (22.0–27.8)	0.151
Baseline liver disease							
HCV:HBV:NASH:others	43:7:13:21	29:5:8:13	14:2:5:8	0.942	21:4:7:10	18:3:4:9	0.938
HCC (+) (%)	29 (34)	21 (38)	8 (27)	0.331	17 (40)	10 (29)	0.316
History of variceal treatment(%)	30 (35)	28 (51)	12 (46)	0.648	24 (50)	10 (35)	0.227
Systolic blood pressure (mmHg)	114 (103–123)	114 (104–125)	111 (99–119)	0.371	114 (101–124)	114 (104–121)	0.916
Diastolic blood pressure (mmHg)	64 (57–70)	64 (57–74)	64 (57–66)	0.197	65 (58–74)	63 (55–67)	0.167
Pulse rate (beats/minute)	76 (69–84)	76 (69–86)	75 (67–82)	0.492	74 (67–84)	77 (71–84)	0.400
Child-Pugh score	11 (9–12)	11 (9–12)	11 (9–12)	0.649	10 (9–12)	11 (9–12)	0.092
MELD	16 (12–20)	15 (12–19)	17 (12–21)	0.352	**15 (12–18)** ^*^	**16 (12–22)** ^*^	**0.033** ^*^
Serum creatinine level	0.75 (0.32–2.88)	0.81 (0.37–2.31)	0.70 (0.32–2.88)	0.183	0.70 (0.37–2.31)	0.86 (0.32–1.77)	0.112
Blood gas analysis							
pH	7.43 (7.41–7.45)	7.43 (7.41–7.45)	7.44 (7.41–7.45)	0.612	7.43 (7.41–7.45)	7.43 (7.41–7.45)	0.693
pO_2_ (mmHg)	83.8 (76.5–97.9)	**93.2 (83.8–108)** ^*^	**73.4 (69.4–77.9)** ^*^	**< 0.001** ^*^	87.0 (78.4–99.6)	82.6 (74.8–97.6)	0.360
pCO_2_ (mmHg)	37.7 (34.3–41.0)	37.6 (34.3–40.9)	38 (34.3–41.9)	0.631	37.4 (34.2–40.6)	38.1 (35.1–41.6)	0.480
HCO_3_^−^ (mmHg)	24.4 (22.6–27.0)	24.5 (22.5–27)	24.4 (23.5–28)	0.434	24.3 (22.5–27.0)	24.6 (22.6–27.5)	0.937
AaDO_2_ (mmHg)	15.8 (6.2–27.0)	**9.4 (−0.6–15.6)** ^*^	**28.4 (24.0–33.4)** ^*^	**< 0.001** ^*^	15.2 (2.8–23.4)	17.2 (5.2–27.9)	0.341
Transthoracic echocardiography							
Left atrial diameter (mm)	38 (36–42)	38 (37–42)	38 (35–44)	0.946	38 (34–40)	39 (37–44)	0.058
Ejection fraction (%)	71 (68–75)	71 (67–75)	73 (69–76)	0.425	70 (66–75)	73 (69–76)	0.093
Cardiac output (L/min)	4.9 (4.2–6.2)	4.7 (4.0–6.3)	5.2 (4.5–6.2)	0.648	5.0 (4.0–6.2)	5.6 (4.5–7.3)	0.303
Left atrial volume index (mL/m^2^)	40 (33–45)	39 (33–45)	41 (31–45)	0.969	41 (33–46)	41 (33–44)	0.950
Tricuspid regurgitation peak gradient (mmHg)	25 (21–30)	24 (20–28)	26 (21–31)	0.371	21 (19–23)	30 (27–36)	–
Left ventricular end-diastolic diameter (mm)	49 (46–52)	48 (45–52)	49 (46–52)	0.713	48 (44–51)	50 (46–54)	0.153
Left ventricular end-systolic diameter (mm)	28 (26–32)	28 (26–31)	28 (26–32)	1.000	28 (26–31)	28 (26–32)	0.910
Interventricular septal thickness (mm)	9 (8–10)	9 (8–10)	9 (8–10)	0.743	9 (8–10)	9 (8–10)	0.326
Right atrial dilatation (n [%])	15 (18)	10 (19)	5 (19)	0.969	7 (16)	8 (23)	0.454
Right ventricular dilatation (n [%])	4 (5)	3 (6)	1 (4)	0.704	**0 (0)** ^*^	**4 (11)** ^*^	**0.022** ^*^
Right heart catheterization							
mPAP-FIO_2_0.6	19 (17–24)	19 (18–23)	21 (17–25)	0.555	18 (17–23)	21 (18–24)	0.114
Perioperative factors							
Perioperative blood loss volume (ml)	4910 (2325–7007)	5095 (2317–7455)	4810 (2325–7007)	0.988	5400 (2762–7690)	4765 (2197–6500)	0.658
Graft recipient weight ratio (%)	0.92 (0.76–1.14)	0.93 (0.80–1.24)	0.91 (0.70–1.12)	0.139	0.99 (0.79–1.17)	0.91 (0.75–1.08)	0.296

*HPS* hepatopulmonary syndrome, *TRPG* tricuspid regurgitation pressure gradient, *BMI* body mass index, *HCV* hepatitis C virus-related cirrhosis, *HBV* hepatitis B virus-related cirrhosis, *NASH* non-alcoholic steatohepatitis-related cirrhosis, *HCC* hepatocellular carcinoma, *PT-INR* prothrombin time international ratio, *MELD* model for end-stage liver diseases; *AaDO*_*2*_ alveolar-arterial oxygen gradient, *mPAP-FIO*_*2*_*0.6* mean pulmonary artery pressure measured after general anesthesia with FIO_2_0.6

^*^p < 0.05

Informed consent was obtained from each patient included in the study, and the study protocol conformed to the ethical guidelines of the 1975 Declaration of Helsinki as reflected in the approval by the ethics committee at Okayama University Hospital. After obtaining the patients’ written informed consent, a detailed medical questionnaire was completed by the doctors.

### Definitions of subclinical HPS and a subclinical high TRPG

All of the patients underwent an arterial blood gas analysis and TTE before OLT as pre-operative screening. The patients were classified as having subclinical HPS when they exhibited elevated AaDO_2_ levels (> 15 mmHg) and decreased PaO2 levels (< 80 mmHg) according to the definition by a European Respiratory Society task force [9]. If a patient exhibited subclinical HPS, perfusion lung scanning using technetium-99 m-labeled macroaggregated albumin ([99 m]Tc-MAA) scintigraphy was performed to diagnose definite HPS. There was only one patient who exhibited definite HPS. The patients were classified as having a subclinical high TRPG when they exhibited a relatively elevated TRPG of ≥25 mmHg according to the median TRPG levels before LDLT. The TRPG data were available from 76 patients. If a patient exhibited a subclinical high TRPG, the peak TR velocity was assessed to select the patients requiring further examinations according to the 2015 ESC/ERS guidelines for the diagnosis and treatment of pulmonary hypertension [7]. There was only one patient who exhibited definite POPH requiring anti-PH treatment.

We performed Swan-Ganz catheterization just before the operation after administering general anesthesia for OLT to determine the mPAP. Although mPAP> 25 mmHg could be diagnosed with definite POPH, the mPAP obtained in the present study was performed under 60% O_2_ with mechanical ventilation and was likely affected by this condition. We used these advisory mPAP data, namely mPAP-FIO_2_0.6, to identify subclinical high TRPG patients in detail.

### Post-OLT management of the subjects

Post-OLT, the patients were treated with a standard immunosuppressive regimen (tacrolimus or cyclosporine A with steroids and/or mycophenolate mofetil). Patients with hepatitis C recurrence were treated with interferon-containing regimens, except for five recently transplanted patients who received direct anti-viral agents.

### Statistical analyses

The JMP software program (Version 13.0.0; SAS Institute Inc., Cary, NC, USA) was used to conduct the statistical analyses. Continuous variables were expressed as the median (interquartile range), and the Mann-Whitney *U*-test or chi-squared test was used to compare the parameters between patients with subclinical HPS and subclinical non-HPS or patients with a subclinical high TRPG and subclinical non-high TRPG. A univariate analysis to define the factors affecting the survival was performed by a log-rank test. The factors found to be significant according to a univariate analysis and the widely accepted post-OLT survival-related factor ‘MELD score’ were used in the multivariate Cox regression analysis. The correlations between the TRPG and mPAP were analyzed using Spearman’s rank correlation method. Statistical significance was set at *P* < 0.05. The four subgroups were compared using the Mann-Whitney *U*-test or chi-squared test with Bonferroni’s correction.

## Results

### General characteristics of subclinical HPS and subclinical high TRPG

The baseline liver diseases were 43 hepatitis C virus (HCV)-related cirrhosis, 7 hepatitis B virus (HBV)-related cirrhosis, 13 non-alcoholic steatohepatitis (NASH), and 21 other etiologies. Twenty-nine patients (34.1%) had hepatocellular carcinoma (HCC) (Table 1). The median model for end-stage liver disease (MELD) score was 16, reflecting decompensated liver cirrhosis. The subclinical HPS patients exhibited no marked clinical differences from the no-HPS patients. However, the subclinical high TRPG patients had a higher MELD score than the low/normal-TRPG patients (*p* = 0.033), indicating a more severe condition of cirrhosis. TTE revealed right ventricular dilatation more frequently in subclinical high TRPG patients than in other patients (*p* = 0.022).

### TRPG and mPAP-FIO_2_0.6

**Fig. 1:**
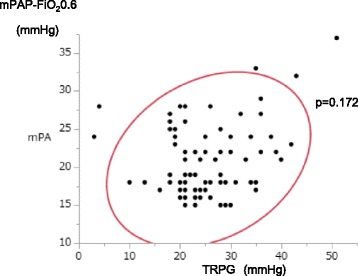
The correlation between TRPG and mPAP-FIO_2_0.6. TRPG; tricuspid regurgitation pressure gradient (TRPG), mPAP-FIO_2_0.6; mean pulmonary artery pressure (mPAP) measured after general anesthesia with FIO_2_0.6

The TRPG levels observed by TTE and mPAP-FIO_2_0.6 levels observed by Swan-Ganz catheterization were not significantly correlated in our study, although they are widely accepted as correlated (Fig. 1). To determine the reason for this discrepancy, the clinical characteristics were investigated according to the TRPG and mPAP-FIO_2_0.6 value (Table 2). Patients with a low/normal-TRPG and low mPAP-FIO_2_0.6 (Group 4; *n* = 34) were most frequently identified, exhibiting the lowest MELD score among the groups examined, as expected. Patients with a high TRPG and high-mPAP-FIO_2_0.6 (Group 1; *n* = 8) with clinically definite PH tended to have a high MELD score (*p* = 0.006 vs Group 4). Patients with a high TRPG and low mPAP-FIO_2_0.6 (Group 2; *n* = 25) did not show right heart dilatation. Patients with a low/normal-TRPG and high mPAP-FIO_2_0.6 (Group 3; *n* = 7) had the highest rate of positive subclinical HPS (*p* = 0.003 vs Group 4), suggesting that the high congestive pressure might be decreased via right-to-left pulmonary shunting.

**Table 2:** Patient characteristics according to TRPG and mPAP status

	Group 1 (*n* = 8)	Group 2 (*n* = 25)	Group 3 (*n* = 7)	Group 4 (*n* = 34)	*p*
TRPG ≥25	yes	yes	no	no	
mPAP-FIO_2_0.6 > 25	yes	no	yes	no	
Age	55 (48–62)	58 (51–61)	59 (54–61)	58 (49–62)	n.s.
Donor age	34 (23–39)	43 (34–56)	38 (33–52)	40 (31–54)	n.s.
MELD	22 (17–25)	16 (12–22)	18 (12–20)	14 (12–17)	0.006 (1 vs 4)
Right atrial dilatation (n [%])	5 (62%)	2 (8%)	3 (42%)	4 (11%)	0.001 (1 vs 2) 0.001 (1 vs 4)
Right ventricular dilatation (n [%])	3 (37%)	1 (4%)	0	0	0.011 (1 vs 2) < 0.001 (1 vs 4)
Subclinical HPS (n [%])	3 (37%)	11 (44%)	5 (71%)	6 (17%)	0.003 (3 vs 4)
Graft recipient weight ratio	1.11 (0.95–1.36)	0.88 (0.62–0.93)	0.81 (0.78–1.19)	0.99 (0.79–1.16)	0.015 (1 vs 2)

*TRPG* tricuspid regurgitation pressure gradient, *mPAP-FIO*_*2*_*0.6* mean pulmonary artery pressure measured after general anesthesia with FIO_2_0.6, *MELD* model for end-stage liver disease, *HPS* hepato-pulmonary syndrome

p: Wilcoxon rank-sum test with Bonferroni’s correction

### Post-LDLT three-month and one-year survival-related factors

The pre- and peri-operative risk factors that correlated with the short-term (three-month and one-year) survival are shown in Table 3. A subclinical high TRPG was the only factor found to be correlated with the three-month survival (*p* = 0.004). A higher donor age and MELD score and a subclinical high TRPG were significantly correlated with the one-year survival. However, a multivariate analysis revealed a subclinical high TRPG to be the only significant factor (p = 0.003). The standard cardiac function as reflected by the ejection fraction was not correlated with the survival or post-LDLT renal function.

**Table 3:** The survival rate and clinical characteristics

	3-month survival-related factors	12-month survival-related factors				40-month survival-related factors			
	Univariate *p*-value	Univariate *p*-value	Risk ratio	95% CI	Multivariate *p*-value	Univariate *p*-value	Risk ratio	95% CI	Multivariate *p*-value
Total									
Recipient age (years)									
≥ 58	0.674	0.389				0.199			
< 58									
Donor age (years)									
≥ 39	0.089	0.065				**0.022** ^*^	**4.14**	**1.41–15.02**	**0.008** ^*^
< 39									
Sex									
Male	0.516	0.507				0.251			
Female									
Baseline liver disease									
HCV	0.874	0.981				0.897			
HBV									
NASH									
Others									
HCC									
(+)	0.255	0.852				0.883			
(−)									
MELD									
≥ 21	0.150	**0.006** ^*^	1.46	0.43–4.59	0.518	**0.005** ^*^	2.23	0.72–6.33	0.153
< 21									
Past history of variceal treatment									
(+)	0.676	0.414				0.619			
(−)									
pO_2_ (mmHg)									
≥ 83.8	0.640	0.324				0.155			
< 83.8									
AaDO_2_ (mmHg)									
≥ 15.9	0.694	0.776				0.489			
< 15.9									
Subclinical HPS									
(+)	0.629	0.274				0.057			
(−)									
Subclinical high-TRPG									
(+)	**0.004** ^*^	**0.001** ^*^	**7.30**	**1.82–48.62**	**0.003** ^*^	**0.002** ^*^	**4.01**	**1.33–14.75**	**0.012** ^*^
(−)									
mPAP-FIO_2_0.6									
≥ 25	0.660	0.152				0.097			
< 25									
Ejection fraction (%)									
≥ 71	0.901	0.461				0.564			
< 71									
Left atrial diameter (mm)									
≥ 38.0	0.432	0.982				0.544			
< 38.0									
Perioperative blood loss volume (ml)									
≥ 4910	0.092	0.242				0.295			
< 4910									
Graft recipient weight ratio									
≥ 0.92	0.258	0.057				0.209			
< 0.92									

*HCV* hepatitis C virus-related cirrhosis, *HBV* hepatitis B virus-related cirrhosis, *NASH* non-alcoholic steatohepatitis-related cirrhosis, *HCC* hepatocellular carcinoma, *MELD* model for end-stage liver disease, *AaDO*_*2*_ alveolar-arterial oxygen gradient, *HPS* hepato-pulmonary syndrome, *TRPG* tricuspid regurgitation pressure gradient, *mPAP-FIO*_*2*_*0.6* mean pulmonary artery pressure measured after general anesthesia with FIO_2_0.6

^*^p < 0.05

### Post-LDLT 40-month survival-related factors

**Fig. 2:**
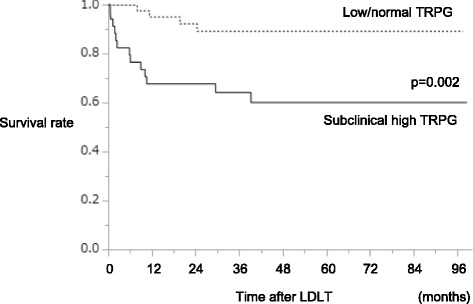
The survival rate according to the existence of subclinical high TRPG. The 40-month survival rate. The solid line indicates a subclinical high TRPG, and the dashed line indicates a non-subclinical high TRPG

The pre- and peri-operative risk factors that correlated with the 40-month survival are shown in Table 3. A higher donor age and MELD score and a subclinical high TRPG were significantly correlated with the 40-month survival after OLT. A multivariate analysis revealed a higher donor age and a subclinical high TRPG to be significant factors (*p* = 0.008 and 0.012 respectively). The survival rate according to the subclinical high TRPG status is shown in Fig. 2. Subclinical high TRPG patients exhibited both a worse early as well as long-term survival after LDLT than the others (*p* = 0.002). The standard cardiac function as reflected by the ejection fraction was not correlated with the survival or post-LDLT renal function, as in the short-term analysis.

### The post-LDLT survival according to TRPG and mPAP-FIO_2_0.6 status

**Fig. 3:**
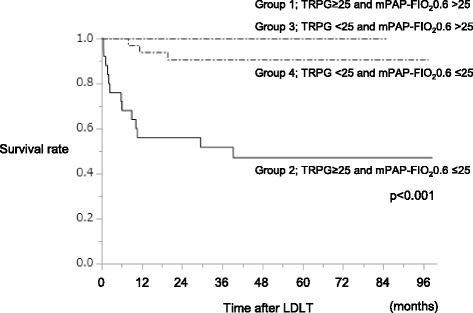
The 40-month  survival rate according to the combination of TRPG and mPAP-FIO_2_0.6. The four groups are grouped according to the combination of TRPG and mPAP-FIO_2_0.6 as in Table 2. Group 1: TRPG≥25 and mPAP-FIO_2_0.6 > 25, Group 2: TRPG≥25 and mPAP-FIO_2_0.6 ≤ 25, Group 3: TRPG < 25 and mPAP-FIO_2_0.6 > 25, Group 4: TRPG < 25 and mPAP-FIO_2_0.6 ≤ 25

Given that a high TRPG but not a high mPAP-FIO_2_0.6 was a significant risk factor for the post-LDLT survival, the post-LDLT survival was investigated according to the TRPG and mPAP-FIO_2_0.6 status in Table 2 (Fig. 3). The post-LDLT survival was worst in Group 2, which had a high TRPG and low mPAP-FIO_2_0.6, while a high mPAP was not found to be a significant risk factor in the present study, despite it being widely accepted as such (*p* < 0.001). Group 3 patients with a high mPAP-FIO_2_0.6 and low TRPG showed a good survival, probably because co-existing HPS released the regurgitation pressure.

### Causes of death in those with a subclinical high TRPG

The causes of death were not significantly different between groups, but the rate of recurrence of HCV was lower in the subclinical high TRPG patients than in the non-subclinical high TRPG patients (2/10 vs. 2/4) (Table 4). Four deaths were recorded in patients without a subclinical high TRPG, including two with HCV-related hepatitis recurrence. Among the patients with subclinical high TRPG, three deaths due to infection, two due to vascular thrombus and HCV recurrence, and one due to small-for-size graft syndrome (graft recipient weight ratio; 0.55%) and primary non-functioning of the graft were recorded. These factors other than HCV recurrence may be associated with congestion of the grafted liver.

**Table 4:** Causes of deaths according to subclinical high TRPG

	Small-for-size graft syndrome	Primary non-functioning	Vascular thrombus	Infection	HCV recurrence	Others
Subclinical high TRPG						
(+)	1	1	2	3	2	1
(−)	0	0	0	1	2	1

*TRPG* tricuspid regurgitation pressure gradient, *HCV* hepatitis C virus

## Discussion

In the present study, a high TRPG was a definite survival-defining factor after LDLT that might be able to be controlled before or during operation. Even in patients with a high mPAP-FIO_2_0.6, the co-existence of subclinical HPS might reduce the TRPG, thus resulting in a good prognosis. The TRPG but not mPAP-FIO_2_0.6 was a critical factor that may reflect the pressure directed to the grafted small living-donor liver. Given that this study was retrospective and the sample size was relatively small, larger prospective studies to confirm the present results are needed.

Reported factors associated with a worse survival after primary DDLT include a high MELD score, HCV positivity, aged recipient, aged donor [10], mechanical ventilation before OLT, and dialysis [11]. Although the present data did not include the hepatic venous pressure gradient (HVPG), which is an accurate reflection of the portal pressure gradient, we included the Child-Pugh score, MELD score and the history of variceal treatment to assess the severity of cirrhosis. As survival-related factors after LDLT, small-for-size graft syndrome is an additional important factor [12]. The present investigation indicated that a pre-LDLT subclinical high TRPG was an independent strong survival-determining factor after LDLT. Although not all patients with a subclinical high TRPG exhibited PH, congestive pressure to the grafted liver likely affected the small living donor graft function recovery post-LDLT.

In LDLT settings, graft blood flow in the initial few weeks after surgery has been reported to influence the overall outcome of small-for-size liver graft OLT. Poor venous outflow and resulting hepatic congestion have been shown to be strong factors for a poor graft function [13]. Several vascular surgical techniques to reduce hepatic venous congestion have been adopted to improve the living donor graft survival [14, 15]. These findings indicate that hepatic venous congestion and the resulting graft congestion are relatively frequent prognosis-defining factors in LDLT, although such congestion is not usually severely problematic in DDLT. The present results suggested that a high mPAP measured after general anesthesia did not affect the outcome post-LDLT; however, a subclinical high TRPG measured by TTE proved to be a strong predictor of a bad outcome. The most frequent causes of deaths in the subclinical high TRPG patients were infection and secondly vascular thrombus. Bacterial translocation from the intestine could be induced by pressure at the graft portal tract resulting in severe infection, and vascular thrombus could also be induced by pressure at the graft. These frequent causes of death in the subclinical high TRPG patients all involved, albeit indirectly, high pressure being applied to the grafts.

TRPG measured by TTE is usually positively correlated with mPAP measured by right heart catheterization [16]. However, several reports have published findings denying such a correlation [17, 18]. One analysis reported that over- and underestimation occurred equally frequently with TTE [19]. Another report stated that TTE underestimated PH more often than catheterization, especially in patients with RV enlargement or right heart failure [20]. However, a different report claimed that severe TR could induce altered right heart hemodynamics, resulting in the overestimation of PH [21]. These conflicting findings are partly because TRPG measures the indirect peripheral pulmonary arterial pressure to the right ventricle and right atrium, while mPAP measures the micro-vessel-level central pulmonary arterial pressure. Our present results indicated no significant correlation between mPAP-FIO_2_0.6 and TRPG. Given that O_2_ administration is an accepted supportive therapy for PH, the present mPAP-FIO_2_0.6 may be lower than the mPAP measured by standard right heart catheterization used as a diagnostic marker for PH. Although the present mPAP-FIO_2_0.6 is different from the mPAP measured by the standard method, all of our patients were in a similar medical condition, and therefore the investigation of the correlation in this population was deemed acceptable. The difference between standard mPAP and mPAP-FIO_2_0.6 cannot be determined. The clinical impact of these values on the post-LDLT survival also differed, as a higher TRPG was significantly correlated with the outcome, while a higher mPAP-FIO_2_0.6 was not. The patients included in our present study exhibited a relatively large left atrium with high AaDO_2_ compared with the normal range observed in the Japanese population, indicating right-to-left shunt predominance with subclinical HPS. The co-existence of subclinical HPS was particularly predominant in Group 3, indicating that TRPG is not high even when mPAP is high. The pressure against the grafted liver is reflected by the TRPG, not by the mPAP, as the pressure may be reduced due to pulmonary arterial bypass with HPS. Given the small sample size, especially in Group 1 and 3, larger studies are needed to confirm this possibility.

HPS is more frequently found than POPH and has been acknowledged as a life-threatening complication; however, this condition can be resolved with OLT [22]. The pathophysiology of this condition is considered to be pulmonary vessel dilatation due to the easily identified vascular dilation-related hormonal changes in liver cirrhosis [1]. As we did not perform TTE with a bubble study, which is the standard sensitive imaging test for identifying intra-pulmonary vascular shunting, we diagnosed only one definite HPS patient by lung perfusion scintigraphy. However, the subclinical HPS patients who clinically exhibited HPS phenomena accounted for 33% of the included subjects. This subclinical HPS condition is frequently accompanied by end-stage liver disease. In our present analysis, the Group 3 patients with a relatively high mPAP and a low TRPG exhibited a higher frequency of subclinical HPS than the other groups. The definite POPH with definite HPS combination has been reported to be associated with a poor 1-year survival rate of 68%, while POPH alone is associated with a 91% survival rate excluding OLT recipients [23]. The post-OLT survival was worse in POPH patients with HPS (5 of 5 dead) than in patients with POPH alone (3 of 3 alive). However, our present results concerning the post-LDLT survival indicated the opposite effect. These patients may not be very sick, as they exhibited only “subclinical” high TRPG and HPS. They may therefore be in good enough health to benefit from HPS, although their mPAP-FIO_2_0.6 values were high. Under these conditions, regurgitation pressure from the pulmonary artery might escape via an intra-pulmonary shunt.

The cardio-pulmonary clinical condition of Group 2, who had a high TRPG and low mPAP, was associated with the worst outcome of the examined groups. This condition is similar to left-to-right shunting or right heart failure although not clinically evident. In addition, the Group 2 patients exhibited a lower frequency of RA dilatation than those in Group 1, showing a high TR pressure without RA dilatation, which indicated a strong pressure directed to the grafted liver. RA dilatation may relieve the direct pressure to the graft.

## Conclusion

TRPG as assessed by TTE may be more useful than mPAP by pre-operational right heart catheterization under FIO_2_0.6 for predicting the post-OLT survival. A high TRPG, even below the limit of PH, may reflect pressure to the grafted liver that might be critical for relatively small-for-size LDLT. Given that this study was retrospective and the sample size was relatively small, larger prospective studies to confirm the present results are needed. When patients with a high TRPG undergo LDLT, the administration of therapies to reduce the TRPG should be considered.

## Abbreviations

[99 m]Tc-MAA: Technetium-99 m-labeled macroaggregated albumin
AaDO_2_: Alveolar-arterial oxygen gradient
DDLT: Deceased donor LT
HBV: Hepatitis B virus-related cirrhosis
HCC: Hepatocellular carcinoma
HCV: Hepatitis C virus-related cirrhosis
HPS: Hepatopulmonary syndrome
LDLT: Living donor liver transplantation
MELD: Model for end stage liver disease
mPAP: Mean pulmonary artery pressure
mPAP-FIO_2_0.6: mPAP measured after general anesthesia with FIO_2_0.6
NASH: Non-alcoholic steatohepatitis
OLT: Orthotopic liver transplantation
PCWP: Pulmonary capillary wedge pressure
PH: Pulmonary hypertension
POPH: Portopulmonary hypertension
RA: Right atrium
RV: Right ventricle
TR: Tricuspid regurgitation
TRPG: Tricuspid regurgitation pressure gradient
TTE: Transthoracic echocardiography
